# Poloxamer/Carboxymethyl Pullulan Aqueous Systems—Miscibility and Thermogelation Studies Using Viscometry, Rheology and Dynamic Light Scattering

**DOI:** 10.3390/polym15081909

**Published:** 2023-04-16

**Authors:** Irina Popescu, Marieta Constantin, Maria Bercea, Bogdan-Paul Coșman, Dana Mihaela Suflet, Gheorghe Fundueanu

**Affiliations:** “Petru Poni” Institute of Macromolecular Chemistry, 41-A Grigore Ghica Voda Alley, 700487 Iasi, Romania

**Keywords:** Poloxamer 407, carboxymethyl pullulan, compatibility, viscoelastic properties, sol–gel transition, dynamic light scattering

## Abstract

Thermally-induced gelling systems based on Poloxamer 407 (PL) and polysaccharides are known for their biomedical applications; however, phase separation frequently occurs in mixtures of poloxamer and neutral polysaccharides. In the present paper, the carboxymethyl pullulan (CMP) (here synthesized) was proposed for compatibilization with poloxamer (PL). The miscibility between PL and CMP in dilute aqueous solution was studied by capillary viscometry. CMP with substitution degrees higher than 0.5 proved to be compatible with PL. The thermogelation of concentrated PL solutions (17%) in the presence of CMP was monitored by the tube inversion method, texture analysis and rheology. The micellization and gelation of PL in the absence or in the presence of CMP were also studied by dynamic light scattering. The critical micelle temperature and sol–gel transition temperature decrease with the addition of CMP, but the concentration of CMP has a peculiar influence on the rheological parameters of the gels. In fact, low concentrations of CMP decrease the gel strength. With a further increase in polyelectrolyte concentration, the gel strength increases until 1% CMP, then the rheological parameters are lowered again. At 37 °C, the gels are able to recover the initial network structure after high deformations, showing a reversible healing process.

## 1. Introduction

Thermally-induced gelling systems have received great attention over the last few decades [1,2]. These materials, which are in liquid state at room temperature and undergo a sol–gel transition upon heating at a temperature close to that of the human body, have proved to be very attractive as injectable drug delivery matrices. Polymers used in thermogelling systems are based on polypeptides (gelatin, elastin), polysaccharides (chitosan and its derivatives, hydroxypropylmethyl cellulose), or synthetic polymers (pluronics, polyesters). These thermosensitive polymers have a particular structure that reflects a fine balance between the hydrophobic and hydrophilic groups [1].

Poloxamers are synthetic poly(ethylene oxide)-poly(propylene glycol)-poly(ethylene oxide) (PEO–PPO–PEO) block copolymers with amphiphilic properties [3]. Among them, Poloxamer 407 (PL), known also as Pluronic F127, with the structure (EO)_100_–(PO)_65_–(EO)_100_ is the most studied triblock copolymer due to its thermogelling behavior at physiologically-relevant temperatures [4]. Due to the interaction between the hydrophobic chain’s segments, the poloxamer macromolecules aggregate into micelles with the increase in the temperature or concentration [5]. The micelles have a spherical hydrophobic core formed by PPO and a hydrophilic corona from PEO segments. At high concentrations (above 15% Poloxamer 407), the micelles are formed first, then with a further increase in the temperature, the remaining PL chains are removed from the solution and inserted into micelles. The micelles are then packed into a face-centered cubic structure, which leads to reversible thermogelation [6,7,8].

The main biomedical application of PL micelles is as drug carriers, these being used to solubilize hydrophobic drugs [9]. In situ thermo-gels based on PL are proposed as drug delivery systems in ophthalmic, rectal, vaginal, nasal or buccal formulations [10,11,12,13,14], or as injectable hydrogels [15,16]. The main limitations for the use of PL thermo-gels are their rapid dissolution in contact with physiological fluids, their modest mechanical strength and bioadhesive properties [9,17]. To overcome these drawbacks, PL has been mixt with water-soluble polymers such as Carbopol, polyvinylpyrrolidone, different polysaccharides [10,11,12,13,14], or PL chains grafted on other polymeric backbones [18,19,20]. Ionic polysaccharides such as carboxymethyl cellulose [21], alginate [12,22], hyaluronic acid [23], chitosan [11], or neutral polysaccharides such as hydroxypropylmethyl cellulose [13] were mixed with PL to obtain thermo-gels with increased bioadhesive properties and mechanical strength. Usually, with the increase in the polysaccharide concentration in the thermo-gel, the sol–gel transition temperature decreases, while the gel strength and mucoadhesive force increase [11,12,21,23]. However, when neutral hydrophilic polysaccharides such as dextran, pullulan, or guar gum are mixed with pluronic, phase separation occurs [24,25,26], similar to the polymeric mixture systems of PEG (the hydrophilic component of the triblock copolymer) with dextran or pullulan [27,28]. 

Pullulan is a linear polysaccharide produced from the yeast-like fungus *Aureobasidium pullulans* by fermentation [28,29], which has become an important industrial source of polymeric materials, economically competitive with natural gums made from marine algae and other plants [30]. Its molecule is composed of repeating maltotriose units (consisting in three α-1,4-linked glucose molecules) linked together via α-1,6-glycosidic bonds. The alteration of α-1,4 and α-1,6 bonds confer chain flexibility and high solubility to the pullulan chains [29,31]. These properties, together with their biodegradability, biocompatibility, lack of mutagenic or carcinogenic effects, availability of reactive sites for chemical modification, good bioadhesive and mechanical properties, make pullulan ideal for biomedical applications in tissue engineering and drug delivery [32,33,34]. 

Although the physical mixture of pullulan and pluronics has been used previously by other authors for intranasal [35] or dermal [25] drug delivery, there are no reports about its use as an injectable formulation. Instead, the carboxylated [36] and carboxymethylated derivatives of pullulan (CMP) [37], frequently used as drug carriers [38,39], were successfully conjugated with heparin [36] or Poloxamer 407 [20], and they showed interesting stimuli-sensitive properties with applications in tissue-engineering. As far as we know, no injectable in situ formulations based on the physical mixture of Poloxamer and CMP have been reported.

The aim of the present work is to design and develop a new injectable gel based on a physical mixture of PL and CMP as a promising platform for the local treatment of damaged skin or articular cartilage. In the first step, we identified the CMP sample with the appropriate degree of substitution to ensure the best miscibility of the two polymers in the aqueous solution. Then, a systematic study on the effect of the addition of CMP on the strength of the thermo-gels while maintaining its self-healing properties was performed.

The thermogelation of poloxamer solution (17%) in the presence of CMP was monitored by rheology and by the tube inversion method. The textural properties and the self-healing abilities of the gels at 37 °C were also evaluated. Dynamic light scattering investigations were performed for a better understanding of the generation and association of the micelles with temperature. In conclusion, the present study provides necessary information regarding the appropriate substitution degree of CMP and the composition of PL/CMP mixture to prepare the best gel formulation. 

## 2. Materials and Methods

### 2.1. Materials

Poloxamer 407 (Mw = 12.6 kDa) (PL) was purchased from Sigma-Aldrich Co. (St. Louis, MO, USA) and used without purification. Pullulan (Mw = 200 kDa) was purchased from Hayashibara Lab Ltd. (Okoyama, Japan). Monochloroacetic acid (MCA), sodium borohydride (NaBH_4_) and isopropyl alcohol were supplied from Sigma-Aldrich Co. (St. Louis, MO, USA). Twice distilled water was used in all the experiments.

### 2.2. Synthesis of Carboxymethyl Pullulan

CMP was obtained according to our previous papers [20,37]. Briefly, 10 g of pullulan and 0.05 g of NaBH_4_ were dissolved in 17.5 mL of distilled water and dispersed under vigorous stirring in 60 mL of isopropyl alcohol. Then, 10 mL of NaOH solution (NaOH/OH molar ratio 0.5/1 or 1/1) was added and left for 30 min at 70 °C. At this temperature, the corresponding amounts of sodium monochloroacetate (MCANa) in 20 mL of water followed by 40 mL of isopropyl alcohol (MCANa: anhydroglucosidic unit of pullulan molar ratio 1:1 or 2.5:1) were added sequentially in three steps. The reaction was continued under stirring at 70 °C for 5 h. The reaction mixture was cooled at room temperature, the aqueous phase was separated from the mixture and dialyzed against distilled water (dialysis bag from Medicell International, England; molecular weight cut-off 12,000 g/mol) for 7 days, until the presence of chlorine ions in the washing water was no longer detected (by checking with 0.1% AgNO_3_ solution). The final product, CMP in the form of sodium salt, was recovered by freeze-drying (−57 °C, 0.03 mbar) using a lyophilizer ALPHA 1–2 LD Christ, Germany. The degree of substitution (DS, number of carboxylic groups per anhydroglucose unit) was determined by conductometric titration according to the method of Constantin et al. [20] and found 0.42 and 0.95.

**Figure 1:**
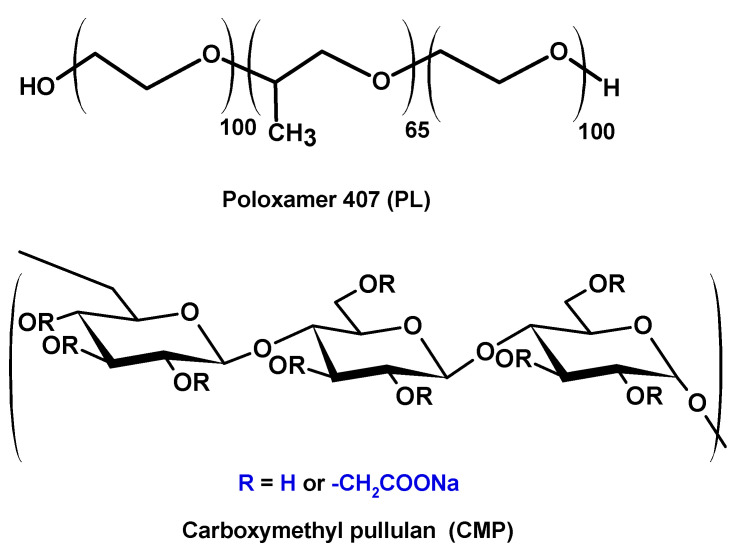
The chemical structure of the polymers.

CMP with a higher DS (DS = 1.6) was obtained by repeating the synthesis procedure starting with carboxymethyl pullulan with DS 0.95. The CMP samples code names are CMP_0.42_, CMP_0.95_, and CMP_1.6_, where the number in subscript represents the substitution degree with carboxymethyl groups. The characterization of pullulan derivatives by conductometric titration and FT-IR spectroscopy (Figure S1) is presented in the Supplementary Materials. The general structures of CMP and PL are presented in Figure 1. 

### 2.3. Miscibility Studies in Dilute Aqueous Solution

The viscometric measurements were carried out at 37 °C with an Ubbelohde viscometer for dilution series with type 0a capillary (diameter of 0.53 mm) and using an AVS 350 Schott automatic viscosity measuring system (Schott, Mainz, Germany). The aqueous stock solutions, 1.5 g/dL PL and 1.5 g/dL CMP, were kept in the refrigerator for 24 h to reach equilibrium, filtered through a sintered glass filter G3 to remove the dust, and then mixed to obtain the desired ratio between the two polymers. The weight fraction of CMP (w_CMP_) was calculated as w_CMP_ = m_CMP_/(m_PL_ + m_CMP_), where m_CMP_ and m_PL_ represent, respectively, the weight of CMP and PL in the polymer mixture. Samples with different weight fractions of CMP_0.42_ and CMP_0.95_ in the polymer mixture were investigated, from w_CMP_ = 0, corresponding to pure PL, to w_CMP_ = 1, corresponding to pure CMP solution. The initial mixture solutions were successively diluted with water inside the viscometer and maintained for at least 20 min at 37 °C for thermal equilibration. Each flow time was measured five times and the mean value was used for the calculation of relative viscosity.

### 2.4. Thermogelation Studies in Concentrated Solutions

#### 2.4.1. Preparation of Formulations

For the thermogelation studies, a stock PL solution (17%, wt:wt) was firstly prepared by the “cold method”: the polymer was added to water under stirring for 4 h on an ice water bath, then maintained at 4 °C for at least 24 h. Formulations containing CMP were prepared by the addition of CMP to this stock solution. The concentration of CMP varied from 0 to 4%. 

#### 2.4.2. Tube Inversion Method

For the first approximation, the sol–gel transition temperature was measured using the tube inversion method [40,41]. Briefly, 1 mL of each formulation in glass vials (12 mm diameter) was heated from 20 °C to 40 °C in a water bath with an 0.2 °C temperature step. At each temperature, the samples were equilibrated for 5 min and their ability to flow was observed by inverting the vials. The sol–gel transition temperature was recorded as the temperature at which the liquid was immobile (no movement of the meniscus over a period of 30 s). The experiments were performed in triplicate.

#### 2.4.3. Evaluation of Gel Hardness

Texture profile analysis (TPA) of the gels was performed using a Brookfield Texture PRO CT3^®^ (Brookfield Engineering Laboratories Inc., Middleboro, MA, USA). The PL solutions introduced into vials (20 mm diameter, wide neck) were thermostated for 1 h at 37 °C using a water bath. The compression was performed at the same temperature, introducing the probe (10 mm diameter) into the gel at defined depth (10 mm) with a speed of 1 mm/s. Three replicate analyses were performed for each formulation at 37 °C. The hardness was determined as the maximum compression force [36,42].

#### 2.4.4. Rheology

The rheological investigations were carried out with a MCR 302 Anton-Paar rheometer (Gratz, Austria) equipped with a Peltier system for temperature control, RHEOPLUS software and plane-plane geometry with a diameter of 50 mm. A gap of 0.5 mm was used for all tests. An anti-evaporation device (Malvern Instruments Ltd., Worcestershire, UK), which creates an atmosphere saturated with solvent in the vicinity of the sample, was used to limit the water evaporation.

The temperature of gelation was determined in oscillatory regime of deformation by following the evolution of viscoelastic parameters as a function of temperature for a heating rate of 0.5 °C/min, in the temperature range 4–50 °C. The viscoelastic moduli, G′ (storage or elastic modulus) and G” (loss or viscous modulus), were determined as a measure of the stored or dissipated deformation energy during one cycle of deformation, respectively. The loss tangent, tan δ = *G″/G′*, expresses the sample’s degree of viscoelasticity. 

The gelation time was determined at 37 °C, using solutions stored in a refrigerator and introduced into the rheometer’s geometry at 4 °C. The temperature was switched at 37 °C at the beginning of the test and the viscoelastic parameters were monitored as a function of time at a constant oscillation frequency (ω) of 1 rad/s and strain amplitude (γ) of 1%. When the gel state was achieved, the thixotropy test was carried out to evaluate the self-healing behavior. The viscoelastic moduli parameters were monitored as a function of time for ω = 1 rad/s and γ successively settled at low 1% and high (values from the nonlinear domain of viscoelasticity) strain amplitude values, respectively.

#### 2.4.5. Dynamic Light Scattering (DLS)

The DLS measurements were performed using a Zetasizer Nano ZS (Malvern Instruments, Malvern, Worcestershire, UK) with a He-Ne Laser (633 nm incident laser wavelength). The back scattering detection system (detection at 173° to the incident beam) used by this instrument allows the measurement of highly concentrated samples because the light does not travel to the entire sample in the cuvette and the multiple scattering phenomenon can be avoided [43]. Using a SOP Player, the temperature was increased from 7 °C to 39 °C with a step of 1 °C, and for each temperature the measurement began after an equilibration time of 10 min. The time-intensity correlation function (ICF), or second-order normalized autocorrelation function, $g^{\left(2\right)}\left(q,t\right)$ was obtained from the instrument and used for further calculations. 

## 3. Results and Discussion

### 3.1. Miscibility Studies in Dilute Solutions

Viscometry is a simple method to study the interactions between two polymers and their compatibility in the presence of a common solvent. This evaluation is based on the deviation of the intrinsic viscosity obtained experimentally for the polymer mixture and the ideal value of this parameter calculated using the additivity law [44,45]. Intrinsic viscosity, [*η*], is a measure of the hydrodynamic volume of macromolecules in the limit of infinite dilution, where the polymer chains are separated from each other. When the solute contains two types of macromolecules, an isolated coil may contain more than one macromolecule [44].

When a polyelectrolyte, such as CMP, is one component of the polymer blend, [*η*] cannot be obtained from the Huggins plots because the reduced viscosity increases exponentially with dilution due to the uncoiling of the charged polymeric chain. For these systems, Wolf approach (Equation (1)) can be applied to obtain the intrinsic viscosity, as it was previously shown for polyelectrolytes or polyelectrolyte/neutral polymer mixtures in aqueous solution without added salt [46,47,48]:

(1) $$\ln\eta_{rel}=\frac{c\left[\eta \right]+Bc^{2}\left[\eta \right]{\left[\eta \right]}^{*}}{1+Bc\left[\eta \right]}$$

where $\eta_{rel}$ is the relative viscosity of the solution, *c* is the concentration (mass per volume), ${\left[\eta \right]}^{*}$ is a specific characteristic hydrodynamic volume (for uncharged molecules ${\left[\eta \right]}^{*}=0$), and *B* is a viscometric interaction parameter corresponding to the Huggins constant. 

**Figure 2:**
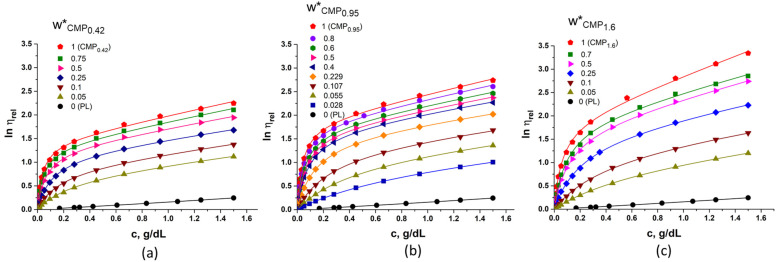
The dependence of $\ln(\eta_{rel})$ on *c* for PL/CMP_0.42_ (**a**), PL/CMP_0.95_ (**b**) and PL/CMP_1.6_ (**c**) in water at 37 °C for different compositions of polymer mixtures. The lines represent the fitting curves according to Equation (1).

On the basis of Equation (1), the values of [*η*] were obtained for polymer mixtures with different PL/CMP compositions by modeling the experimental data of $\ln\eta_{rel}$ as a function of concentration. Figure 2 shows the experimental data and the fitting curves of $\ln\eta_{rel}$ versus *c* for three different systems: PL/CMP_0.42_, PL/CMP_0.95_ and PL/CMP_1.6_. 

**Figure 3:**
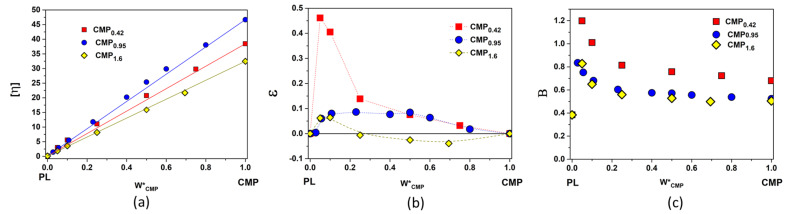
Intrinsic viscosities obtained for PL/CMP_0.42_, PL/CMP_0.95_ and PL/CMP_1.6_ mixtures at 37 °C—the lines represent ideal intrinsic viscosities calculated according to Equation (2) (**a**). The variation of parameter ε (**b**) and parameter *B* (**c**) with the composition of the mixtures (obtained at 37 °C).

The extended conformation in solution of the pullulan derivatives due to the electrostatic repulsion between the charges led to higher values of viscosity of the poloxamer solutions containing CMP. The viscosity in solution of pure PL is very low due to its low molecular weight. However, it should be mentioned that at 37 °C, coiled unimers exist at low concentrations, whereas the micelles are formed at higher concentrations. Being a neutral polymer, the Wolf plots for poloxamer present a linear dependence. On the other side, solutions of CMP and their mixtures with PL show typical polyelectrolyte behavior. The obtained values of [*η*] for the three systems PL/CMP_0.42,_ CMP_0.95_ and PL/CMP_1.6_, are presented in Figure 3a. The value of [*η*] for pure CMP_0.95_ was higher than that of CMP_0.42_ due to the increased charge density. Unexpectedly, for pure CMP_1.6_, the intrinsic viscosity was lower than for the other two derivatives. The decrease in [*η*] is probably related to the decrease in molecular mass caused by the degradative synthesis procedure, since CMP_1.6_ was obtained by the carboxymethylation of pullulan in two steps.

Considering the coexistence of isolated CMP coils and isolated poloxamer coils in dilute solution, the ideal intrinsic viscosity of the polymeric blend, $\bar{\left[\eta \right]}$, is calculated as (Equation (2)):

(2) $$\bar{\left[\eta \right]}=w_{CMP}^{*}{\left[\eta \right]}_{CMP}+w_{PL}^{*}{\left[\eta \right]}_{PL}$$

where $w_{CMP}^{*}$ and $w_{PL}^{*}$ are the weight fractions of the components in the mixture, ${\left[\eta \right]}_{CMP}$ and ${\left[\eta \right]}_{PL}$ are, respectively, the intrinsic viscosity of the two polymers alone in their aqueous solution. The deviation from the ideality of the experimental values of [*η*] determined for the polymer mixture can be expressed using a parameter ε defined as:

(3) $$\varepsilon =\frac{\left[\eta \right]-\bar{\left[\eta \right]}}{\bar{\left[\eta \right]}}$$

The absence of interactions between the two polymers leads to an ideal behavior and ε is close to 0. Negative values of parameter ε reflect the favorable interaction between the segments of the two unlike polymers when the hydrodynamic volume of the mixt coil is smaller than in ideal conditions (an isolated coil can contain two unlike molecules) [44]. Positive values of parameter ε mean that the hydrodynamic volume of both polymers in the mixture is larger than the sum of those corresponding to the two binary isolated macromolecular coils [44,49,50]. 

According to Figure 3b, for the PL/CMP_0.42_ and PL/CMP_0.95_ mixtures, the parameter ε > 0 over the whole composition range. This means that the interaction between PL and CMP chains determines the increase in the hydrodynamic volume of the isolated coils, or the existence of mixed coils of CMP and PL with increased dimensions. This deviation is more pronounced for CMP_0.42_ in the presence of high fractions of PL. Such behavior was also obtained for other polymeric mixtures with different hydrophobic-hydrophilic character such as PL/poly(aspartic acid) [51], poly(vinyl alcohol)/bovine serum albumin [50], or poly(vinyl alcohol)/poly(urethane) [52]. For PL/CMP_1.6_ mixtures, at high fractions of PL, the parameter ε is also positive. However, with the increase in CMP_1_._6_ fraction, this parameter decreases, reaching even slightly negative values; this means that the deviations from the ideal hydrodynamic volume are very low and some interactions between the two polymers take place. A similar behavior was observed for the PL/hydroxypropyl cellulose aqueous mixtures at 37 °C [45].

If the parameters [*η*] and ε offer information about the isolated coils, the parameter *B* from the Equation (1) is related to the deviation of $\ln\eta_{rel}$ versus c from the linear dependence on the entire studied concentration domain (0–1.5 g/dL). This parameter quantifies the viscometric interaction between the polymer segments. It can offer information about the quality of the solvent: *B* has positive values in thermodynamically good solvents and negative values in sufficiently unfavorable solvents [53]. Figure 3c presents the variation of this parameter with the composition in PL/CMP mixtures. It can be observed that *B* passes through a pronounced maximum at low fractions of CMP, which signifies the least probability for the formation of intersegmental polymer contact. In other words, in the presence of high fractions of PL, the polymeric chains have an increased interaction with water. The *B* value is higher for PL/CMP_0.42_ in pure water compared to PL/CMP_0.95_ or PL/CMP_1.6_, a tendency found also for other cationic derivatives of dextran [54]. This phenomenon is related to the substitution degree of CMP. Higher DS of CMP means a more extended chain conformation in solution and higher probability to interact to PL chains (micelles). The *B* values for the PL/CMP_1.6_ system were expected to be lower than those for the other two mixtures due to the increased interaction between components. However, these values were close to those of the PL/CMP_0.95_ system (Figure 3c) and the similarity could be explained by the lower molecular weight of the CMP_1.6_ derivative. As it is known, the *B* parameter increases with the decrease in the molecular weight of the polyelectrolyte [55], confirmed by the smaller values of [*η*] for CMP_1.6_ (Figure 3b). Once the maximum is passed, the *B* values decrease almost linear with the increase in CMP fractions in the polymer mixture until w*_CMP_ ≅ 0.3 and then remained constant. This behavior can be attributed to the prevalence of the thermodynamic favorable contacts between polymer segments over contacts between solvent molecules and polymer segments; in these conditions the viscosity increase occurs via the formation of a weak physical network and leads to lower *B* values [54]. Furthermore, with the increase in CMP concentration, the electrostatic shielding of the charged groups and, hence, the shrinkage of the chains, takes place.

**Figure 4:**
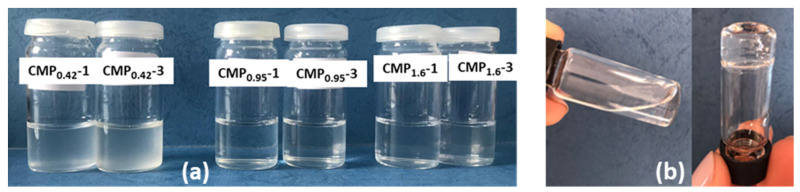
(**a**) Concentrated poloxamer solution (17%) with 1% and 3% added CMP_0.42_, CMP_0.95_, and CMP_1.6_ (from left to right) at room temperature. (**b**) Tube inversion method for 17% poloxamer with 1% CMP_0.95_ below (**left**) and above (**right**) the gelation temperature.

When CMP_0.42_ is added to the concentrated PL solution (17%), a phase separation occurs and turbidity appears (Figure 4a). The phase separation that appears in pullulan/PEO, dextran/PEO mixtures or other aqueous two-phase systems can be explained from two points of view: (i) energetically unfavorable segment–segment interactions of polymers overcome the entropy increase involved in phase separation, or (ii) another key factor of phase separation is the structure of water around the polymeric chains, meaning that the two polymers form different polymer-specific water hydrogen bond domains with dissimilar solvent properties, and in concentrated solutions, these domains are immiscible [56,57,58]. 

The observation of the phase separation in concentrated solutions of PL with CMP_0.42_, together with the high values of the parameters ε and *B* for PL/CMP_0.42_ diluted mixtures (Figure 3b,c), shows the incompatibility between these two polymers at high fraction of PL. Increasing the substitution degree of pullulan with carboxylic groups leads to a better miscibility with PL. The introduction of ionic groups on the pullulan chains brings ion-dipol interactions with water molecules for solubilization, so the water structure around CMP chains is modified. The lack of chemical interactions between CMP and PL was demonstrated by FTIR spectroscopy (Supplementary material, Figure S2), but the ionized carboxylic groups from CMP can interact trough Na^+^ ion bridges with the –OH groups from PEO [47] or through hydrogen bonds between the undissociated -COOH groups and the oxygens from the poloxamer chains [51,59,60]. Therefore, the mixture of 17% PL with CMP_0.95_ or CMP_1.6_ does not present turbidity (Figure 4a), regardless of the temperature. Consequently, only these two pullulan derivatives were used in further experiments.

### 3.2. Gelation Temperature

**Figure 5:**
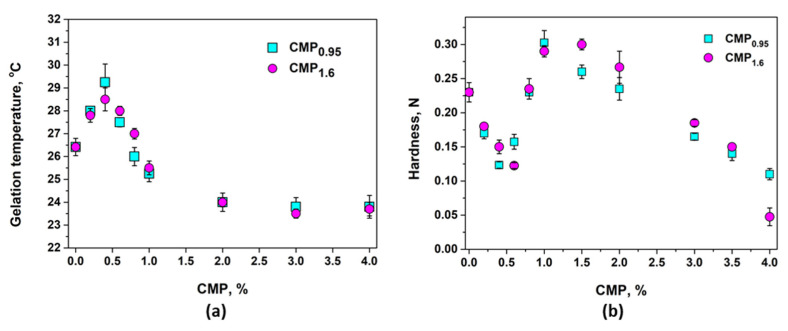
Variation in the gelation temperature (determined by test inversion method) (**a**) and of the gel hardness (texture analysis method) (**b**) of 17% PL solution with the addition of CMP in different concentrations.

The thermogelation of 17% PL solution in the presence of CMP_0.95_ or CMP_1.6_ in different concentrations was investigated. In a first approximation, the sol–gel transition temperature was determined by the tube inversion method [26,40] (Figure 4b). This method makes it possible to measure the temperature required for the formation of hard gels, and it is different from the sol–gel temperature determined by rheology [26,40,41]. From Figure 5a, which presents the effect of CMP addition on the gelation temperature, it can be observed that CMP_0.95_ and CMP_1.6_ have almost the same effect. Low concentrations of polyelectrolyte (below 0.5%) determine an increase in the gelation temperature, a further increase in the CMP concentration, leading to a reduction of it, as expected [11,14,21,61]. When 5% CMP was added, a very soft gel was formed displaying flow properties, and the gelation temperature could not be measured by the tube inversion method. This behavior could be explained by the modest viscosity of CMP due to its relatively low molecular mass and high chain flexibility, compared with other ionic polysaccharides.

It is known that the addition of polymers such as sodium carboxymethyl cellulose [21,62], gellan gum [63], sodium alginate [14] or chitosan [11] increases the viscosity of poloxamer solution and decreases the gelation temperature. Despite this, there are also studies showing that Carbopol 971P, Polycarbophyl, or xanthan gum at low concentrations increase the gelation temperature of pluronic solution, and at high concentrations decrease this temperature [26,64,65]. 

### 3.3. Gel Hardness

The influence of CMP addition on the hardness of poloxamer gels obtained at 37 °C is presented in Figure 5b. The hardness of the 17% poloxamer gels decreases with the addition of low concentrations of CMP (below 0.5%), then increases, with a maximum at around 1% polyelectrolyte being observed. Above this concentration, the addition of CMP decreases the hardness of the gel. Generally, the addition of polyelectrolytes such as chitosan, sodium carboxymethylcellulose, sodium alginate, and poly(acrylic acid) [11,12,62,66] leads to an increase in poloxamer gels hardness; however, a similar trend in gel hardness formed at 37 °C of mixture of Carbopol 971P or Polycarbophil and Poloxamer 407 (15%) was found by De Souza Ferreira [64,65]: a decrease at low concentration of polyelectrolyte, an increase at 0.15% polyelectrolyte, then again a decrease with a further increase in polyelectrolyte concentration. Furthermore, the same peculiar behavior was observed for CMP_0.95_ as for CMP_1.6_ due to their relatively high charge density and probably similar configuration in solution. In further studies, only the influence of CMP_0.95_ was investigated by rheology and DLS.

### 3.4. Rheological Behavior

All studies concerning the thermogelation behavior of poloxamer solution (17%) in the presence of CMP were realized for the formulations presented in Table 1.

**Figure 6:**
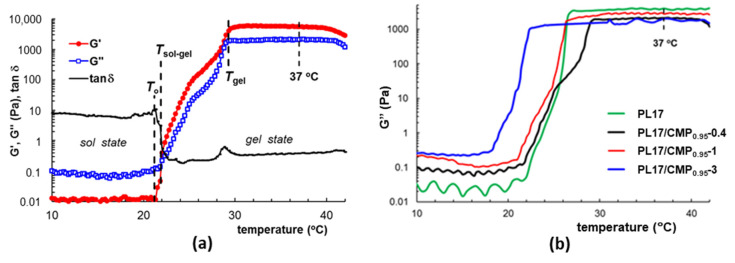
Sol–gel transition illustrated for sample PL17/CMP_0.95_-0.4 through the dependence of the viscoelastic parameters on temperature (**a**); variation of loss modulus during temperature induced gelation for PL17/CMP_0.95_ samples (**b**) (heating rate of 0.5 °C/min, ω = 1 rad/s, γ = 1%).

Figure 6 presents the sol–gel transition induced by temperature increase by following the variation in rheological parameters. According to Figure 6a, the sol state is clearly evidenced by the small values of viscoelastic moduli G′< G″ and tanδ > 1. Below the temperature *T*_o_, which marks the onset of gelation, the PL unimers and micelles coexist in solution with CMP chains and the temperature increase has a small influence. Above *T*_o_, when the temperature rises by less than 10 °C, the viscoelastic moduli suddenly increase within several decades and the increase in G′ is faster than G″. This is due to structural changes induced by temperature, from micelles to polymicelles and network structure. At the transition point from sol state to gel state, denoted *T*_sol–gel_ (which is close to *T*_o_), G′ = G″ and tanδ = 1. G′ > G″ above this temperature and around *T*_gel_ the network is formed and the equilibrium is reached. The samples behave differently when CMP_0.95_ is added in the PL system (Figure 6b), and the temperatures *T*_o_ and *T*_sol–gel_ are shifted to lower values (Table 2), as expected from the literature [11,21,62,63]. The addition of a small amount of CMP decreases the transition temperatures and disturbs the gelation kinetics and PL network structure. 

With the increase in the CMP concentration, T_sol–gel_ decreases, but T_gel_, which is in good accordance with the T_gel_ measured by the tube inversion method (temperature required for hard gel formation), shows a peculiar behavior: first it increases (sample PL17/CMP_0.95_-0.4), then decreases. The gel hardness and the rheological parameters (the complex viscosity and viscoelastic moduli) in gel state showed the same tendency: a pronounced decrease with the addition of CMP in low concentration, an increase with the further increase in CMP concentration until 1% CMP, then a decrease again (Table 1 and Figure 5b). 

**Figure 7:**
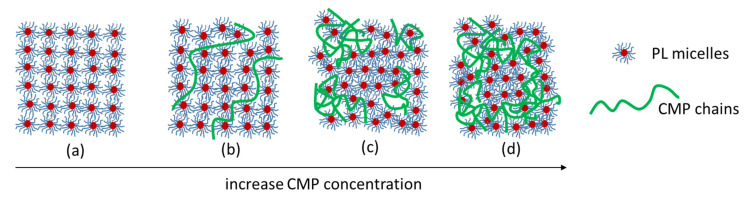
Schematic representation of the influence of CMP concentration on the PL17/CMP gelation behavior: PL17 (**a**), PL17/CMP_0.95_-0.4 (**b**), PL17/CMP_0.95_-1 (**c**) and PL17/CMP_0.95_-3 (**d**).

The CMP has two different effects on gel formation: (i) it increases the concentration of PL in the polyelectrolyte-free domains (that is why the critical micelle temperature (CMT) and the T_sol–gel_ decreases with the increase in CMP concentration), (ii) it perturbs the aggregation of PL micelles into a face-centered cubic structure in the whole network (that is why the rheological parameters of the gels in the presence of CMP are lower compared to PL17). As presented in Figure 7, the polyelectrolyte has an extended conformation at low concentrations, dividing the PL network in many domains and decreasing the gel strength (Figure 7b). With the increase in CMP concentration, the conformation of polysaccharide becomes more coiled, forcing the agglomeration of PL micelles in large macrodomains. Thus, the interactions between micelles are stronger, so the strength of the PL network increases until 1% CMP concentration (Figure 7c). With the further increase in CMP concentration, the flexibility of the gel increases due to the progressive isolation of PL micelles and increasing contribution of CMP (Figure 7d), and the gel rheological parameters decrease. 

In the literature, it was shown that if the addition of xanthan gum with high viscosity onto 17% PL solution increases the viscosity of the gel at 37 °C, the addition of agar gum with low viscosity decreases the viscosity of the gel [26]. This means that the viscosity of the polyelectrolyte has an important influence on the rheological properties of the PL thermogels. 

**Figure 8:**
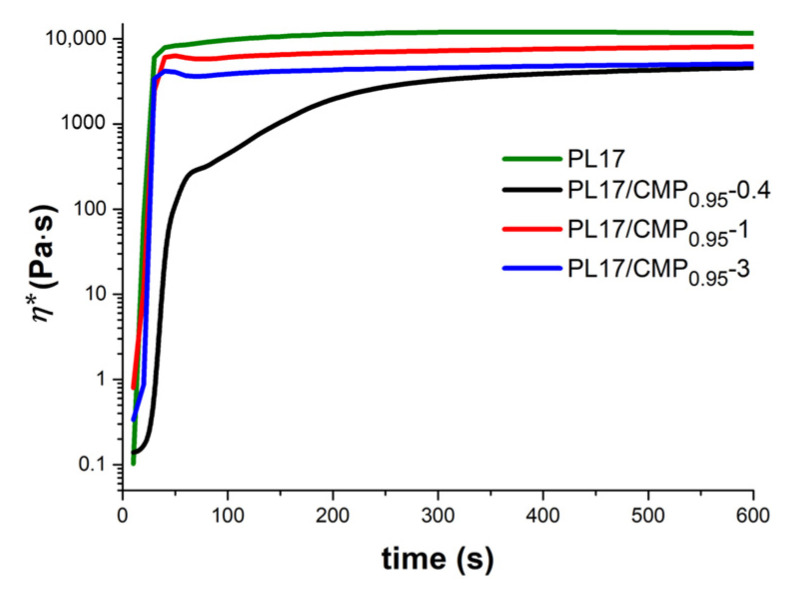
Complex viscosity of PL/CMP mixtures as a function of time when the temperature was switched from 5 °C to 37 °C (ω = 1 rad/s, γ = 1%).

The kinetics of gelation were followed at a constant temperature of 37 °C by monitoring the viscoelastic behavior in time. The samples stored in the refrigerator were poured on the lower plate of the rheometer thermostated at 5 °C and then the temperature was switched at 37 °C. The gel formation is very fast: 30 s for all the samples with the exception of PL17/CMP_0.95_-0.4, where the gelation (G′ > G″) takes place in about 70 s. For PL17/CMP_0.95_-0.4, the equilibrium structure was reached after about 300 s, as it is observed from the variation in the complex viscosity during gelation (Figure 8). This also suggests that a small amount of CMP_0.95_ added to PL solutions disturbs the micelle organization when the temperature increases at 37 °C.

### 3.5. Dynamic Light Scattering Studies

DLS, also known as photon correlation spectroscopy, was usually used to study the micelles formation and micelles hydrodynamic radius in dilute or semidilute pluronic copolymers solutions [67,68,69,70]. Recently, this method was used to investigate the aggregation behavior of pluronic in concentrated solution (20%) without and with added chitosan (0.06%) and montmorilonite [71].

DLS can be also used to investigate the formation of chemically or physically cross-linked gels, as shown by Martin and co-workers [72], Shibayama and co-workers [73,74,75], or other authors [76,77]. The results obtained by this technique are well correlated with the rheological measurements [75,77]. Generally, the gelation threshold was characterized by the appearance of a power-low in the intensity-time correlation function (ICF), a suppression of the initial amplitude of ICF and a speckle pattern in the scattering intensity.

**Figure 9:**
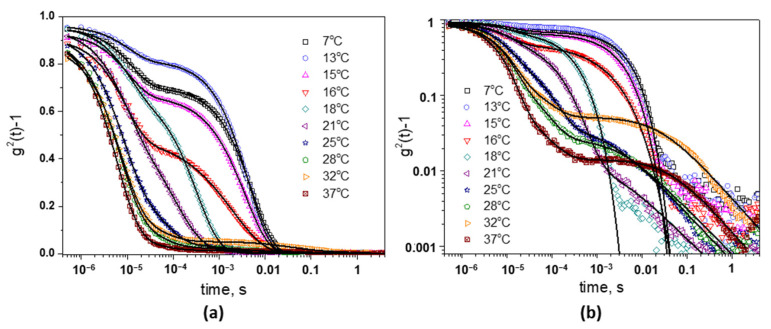
Intensity autocorrelation functions for 17% aqueous Poloxamer 407 solution at different temperatures measured at scattering angle of θ = 173°: g^2^(t)^−1^ vs. log(t) representation (**a**), and log−log representation (**b**). The lines are the fits with Equations (4) and (5).

In this study, DLS measurements of the 17% poloxamer solution, without and in the presence of CMP_0.95_ in different concentrations, were performed. The effect of the temperature increases in the range 7–39 °C on the ICF was investigated. Figure 9a presents the intensity correlation functions for poloxamer 17% solution at different temperatures. At low temperatures (7–15 °C), two inflection points in the representation of $g^{\left(2\right)}\left(q,t\right)-1$ vs. *log (t)* can be clearly evidenced, showing the existence of a two-step relaxation. The fast relaxation mode, in the time scale/region 10^−6^–10^−4^, is attributed to the diffusion of the small poloxamer chains—unimers. The slow relaxation mode can be attributed to the hindered motion of interacting polymeric chains in concentrated solution [78] and it is also observed for pluronic solutions in semidilute or concentrate regime, when the chains are overlapped and entangled [70,71].

When the temperature increases above 15 °C, the shape of the ICF starts to modify, and the second relaxation mode moves to faster relaxation times due to the formation of the micelles. The critical micelle temperature (CMT) for 17% Poloxamer 407 solution is around 16 °C, a value similar to that found in the literature [6,79,80]. At higher temperatures, especially over 21 °C, the appearance of a large shoulder can be observed on the time scale in the range 10^−4^–1 s. This long-time tail was better evidenced in the log−log representation of the ICF (Figure 9b) and can be ascribed to the formation of large aggregates from interacting micelles or to the frozen inhomogeneities of the gel from the point of view of micelles mobility [74].

**Figure 10:**
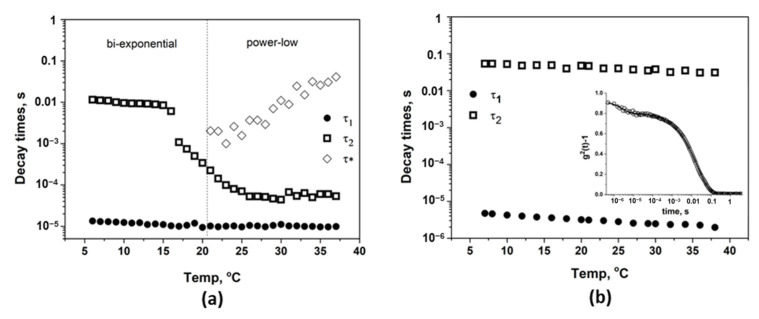
Temperature dependence of the decay times for 17% poloxamer solution (**a**) and for 1% CMP_0.95_ solution (**b**). The intensity autocorrelation function of CMP_0.95_ solution (1%) at 20 °C is presented in the inset (**b**).

In order to describe the presence of two relaxation modes at low temperatures, the ICF can be written as a combination of a single exponential function (the fast mode) and a stretch exponential function (the slow mode) [71,73]:

(4) $$g^{\left(2\right)}\left(t\right)-1=\sigma_{1}^{2}{\left\{\left(1-A_{2}\right)exp\left(-\frac{t}{\tau_{1}}\right)+A_{2}exp\left[-{\left(\frac{t}{\tau_{2}}\right)}^{\beta }\right]\right\}}^{2}$$

where $\sigma_{1}^{2}$ is the initial amplitude of ICF, $\tau_{1}$ and $\tau_{2}$ are the relaxation times characterizing the fast and the slow relaxation process, *A*_2_ (0 < *A*_2_ < 1) is the amplitude/fraction of the slow mode, and *β* (0 < *β* ≤ 1) is the stretched exponent. This equation fits very well with the experimental data from 7 °C to around 20 °C, with the exception of the points of the end of the curves that are considered residuals. The long time tail that appears at higher temperatures can be described by the introduction of a power-low decay [72]:

(5) $$g^{\left(2\right)}\left(t\right)-1=\sigma_{1}^{2}{\left\{\left(1-A_{2}-A_{3}\right)\exp\left(-\frac{t}{\tau_{1}}\right)+A_{2}exp\left[-{\left(\frac{t}{\tau_{2}}\right)}^{\beta }\right]+A_{3}\frac{1}{{\left(1+t/\tau^{*}\right)}^{D_{p}/2}}\right\}}^{2}$$

where *A*_3_ is the amplitude of the power-low tail, $\tau^{*}$ is the time at which the power-low tail begins, and $D_{p}\;$ is the fractal dimension of scatter photons. This equation fits the experimental intensity correlation functions for poloxamer solution at temperatures T ≥ 21 °C (Figure 9b). The decay times obtained by fitting the experimental data with the Equations (4) and (5) are presented in Figure 10a. The fast relaxation process is almost independent of the temperature, but the amplitude of this mode is higher at low temperatures where the unimers are predominant. With the increase in the temperature above 15 °C, unimers are also present in the solution, but their concentration decreases in the favor of the micelles [6]. Even after the complete micellization, the unimers are continuously released and reabsorbed into the micelles [71,81]. The size of the unimers is around 3 nm. 

The decay time of the slow relaxation mode, $\tau_{2}$, drops suddenly above 16 °C, showing the formation of the micelles. With a further increase in the temperature, the micelles shrink due to both the dehydration of the PPO core and of the PEO corona [6,69]. That is why $\tau_{2}$ moves to faster decay times. The size of the micelles was reduced from 250 nm at 17 °C to around 40 nm at 25 °C. At this concentration, the micelles organize themselves into close-packed configuration, so above 26 °C, when the gel becomes macroscopically immobile, $\tau_{2}$ remains almost constant. With a further increase in the temperature, the time at which the power-low tail begins (τ*) moves to longer times and the amplitude of the power-low tail (*A*_3_) rises from 0.1 at 21 °C to around 0.25, showing that the increasing physical interactions between the micelles restricts the movement of the system.

In order to study the behavior of both polymers (the block copolymer and polyelectrolyte), DLS experiments with increasing temperature were also performed for a 1% aqueous solution of CMP_0.95_ (Figure 10b-inset). Two relaxation modes were observed for CMP_0.95_, as expected for a polyelectrolyte in salt-free solution [78]. The fast mode can be interpreted in this case as “mutual diffusion” (coupled diffusion of individual polyelectrolyte chains and of their counter-ions), and the slow mode can be interpreted as “collective diffusion” (diffusion of the center of the mass of the polyelectrolyte chains under the constraints induced by electrostatic interactions with other surrounding chains) [78]. The FIC curves kept the same shape with the increase in the temperature. The decay times obtained by fitting the curves with Equation (4) are presented in Figure 10b. The decay times of the fast mode (5 × 10^−6^–2 × 10^−6^) and of the slow mode (5 × 10^−2^–3 × 10^−2^) are practically temperature independent.

**Figure 11:**
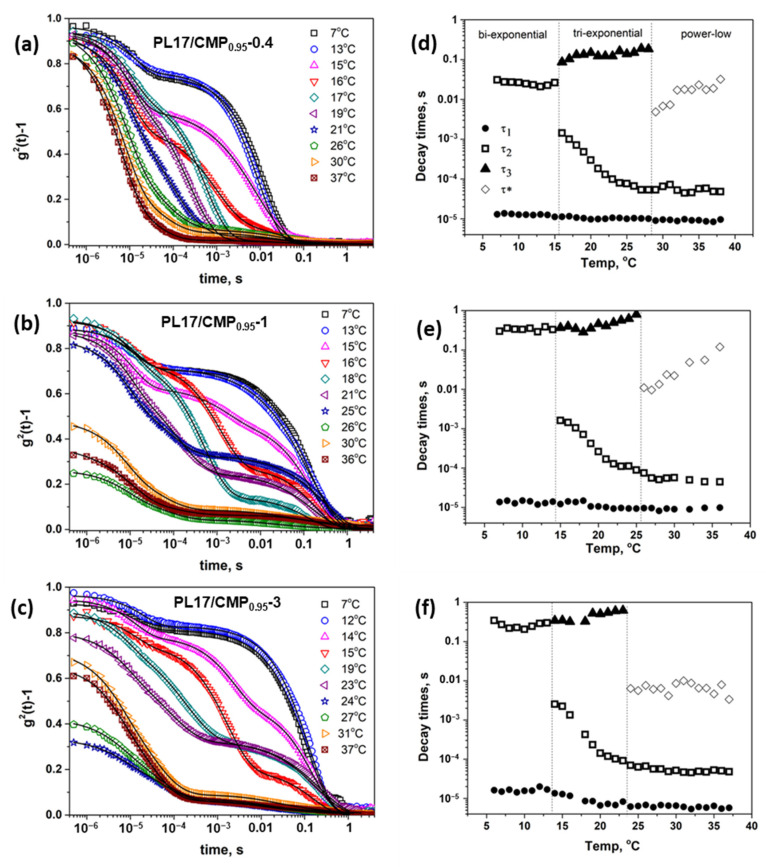
Intensity autocorrelation function for PL17/CMP_0.95_-0.4 (**a**), PL17/CMP_0.95_-1 (**b**) and PL17/CMP_0.95_-3 (**c**). The lines are the fits. Temperature dependence of the decay times for the corresponding systems (**d**–**f**).

Starting from these considerations, the dynamical behavior of poloxamer in the presence of different concentrations of CMP_0.95_ was then analyzed. Figure 11a–c present the intensity correlation functions $g^{\left(2\right)}\left(q,t\right)-1$ at different temperatures for PL17/CMP_0.95_-0.4, PL17/CMP_0.95_-1 and PL17/CMP_0.95_-3. Compared to PL17, it can be observed that the slow relaxation mode becomes slower with the increase in the polyelectrolyte concentration, probably because the movement of the CMP_0.95_ chains is hindered by the total viscosity of the system. The shape of the ICF curves changed when the temperature is around the critical micelle temperature, as in the case of PL17. After the formation of the micelles (above 14–16 °C), three relaxation processes are observed: a fast relaxation due to the diffusion of poloxamer unimers and to the “mutual diffusion” of the polyelectrolyte, a second relaxation ascribed to the diffusion of the micelles, and the slowest mode attributed to the “collective diffusion” of the polyelectrolyte. For the systems with high concentrations of CMP_0.95_ (PL17/CMP_0.95_-1 and PL17/CMP_0.95_-3), a decrease in the initial amplitude of ICF can be observed when the temperature reached the gel temperature determined by the tube inversion method. Above this temperature, the shape of the last relaxation mode was transformed from a stretched exponential to a power-low tail.

Bellow the critical micelle temperature, the IFCs were well fitted by a sum of a single exponential and a stretched exponential, according to Equation (4), but after the formation of the micelles, an additional long time stretched exponential was added to fit the ICFs:

(6) $$g^{\left(2\right)}\left(t\right)-1=\sigma_{1}^{2}{\left\{\left(1-A_{1}-A_{2}\right)exp\left(-\frac{t}{\tau_{1}}\right)+A_{2}exp\left[-{\left(\frac{t}{\tau_{2}}\right)}^{\beta }\right]+A_{3}exp\left[-{\left(\frac{t}{\tau_{3}}\right)}^{\gamma }\right]\right\}}^{2}$$

Above the gel temperature, the long-time stretched exponential was replaced with a power-low decay according to Equation (5). The obtained decay times are represented in Figure 11d–f. If the fast relaxation decay is almost time independent, the drop in $\tau_{2}$ shows the micelle formation. CMT decreases from 16 °C in PL17/CMP_0.95_-0.4 to 15 °C in PL17/CMP_0.95_-1 and 14 °C in PL17/CMP_0.95_-3 system (Table 2). This may probably be due not to the involvement of CMP_0.95_ in the hydrophobic interactions of PPO blocks, but to the increase in poloxamer concentration in the polyelectrolyte free micro-domains. The appearance of the power-low tail occurs at 29 °C for PL17/CMP_0.95_-0.4, at 26 °C for PL-17/CMP_0.95_-1 and at 24 °C for PL-17/CMP_0.95_-3, temperatures at which the gels become macroscopically immobile (Table 2).

### 3.6. Self-Healing Behavior

**Figure 12:**
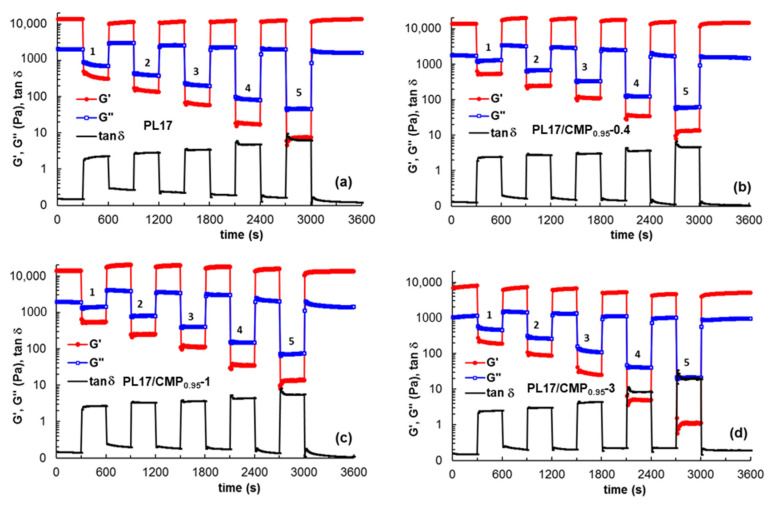
Self-healing behavior for samples PL17 (**a**), PL17/CMP_0.95_-0.4 (**b**), PL17/CMP_0.95_-1 (**c**) and PL17/CMP_0.95_-3 (**d**) by applying step strains cycles of small—high—small strain values. The small level of strain was always 1% and the high level of strain was increased successively: 1–50%; 2–100%; 3–200%; 4–500%; and 5–1000%.

The self-healing behavior, i.e., the ability to recover the structural integrity after applying a mechanical stress, is a requirement for injectable systems used in tissue engineering or drug delivery applications [60,82,83]. Viscoelastic behavior as a function of time during cycles of low-high-low deformations is usually investigated for such materials. In the present study, the thixotropic behavior was analyzed for the PL-based samples and the results are given in Figure 12. Low strains of 1% were alternated every 300 s with increasing high deformations for 5 cycles: 1–50%; 2–100%; 3–200%; 4–500%; and 5–1000%.

For all samples, there is a high degree of recovery showing the ability of the micellar structure to be rapidly reestablished once the external stress is removed. However, particular behaviors can be depicted by calculating the degree of structure recovery after each strain cycle as:

(7) $$R\;(\%)=\frac{\mathrm{elastic}\;\mathrm{modulus}\;\mathrm{before}\;\mathrm{the}\;\mathrm{first}\;\mathrm{cycle}}{\mathrm{elastic}\;\mathrm{modulus}\;\mathrm{after}\;\mathrm{cycle}\;n}\;\times \;100$$

**Figure 13:**
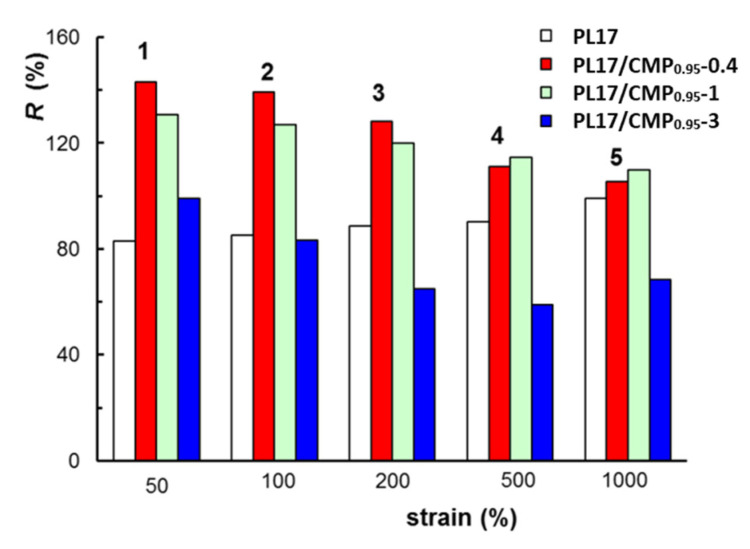
The degree of structural recovery after applying step strains cycles.

According to Figure 13, for samples PL17/CMP_0.95_-0.4 and PL17/CMP_0.95_-1, the network structure is strengthened after removing the applied strains, suggesting an increase in intermolecular interactions and structure reorganization, possibly due to changes in conformation under the action of mechanical forces. By increasing the applied strain, these interactions are disturbed and *R* value decreases from 143% after cycle 1 to 99% after cycle 5. For PL17, an increase in *R* is observed during the 5 cycles of deformation from 83% to 99%, but the *R* value does not exceed 100%. Sample PL17/CMP_0.95_-3 is not able to recover its structure after high deformations, with the *R* value decreasing from 99% after cycle 1 to about 60% after cycle 4 and 5.

## 4. Conclusions

The viscometric studies in dilute aqueous solution showed that CMP chains are in a more extended conformation in the presence of PL. The interactions between the segments of CMP_0.42_ and PL are lower compared to the interaction between CMP_0.95_ or CMP_1.6_ and PL. In concentrated solution, PL/CMP_0.42_ systems present phase separation, while CMP with higher substitution degrees (DS = 0.95 or 1.6) are miscible with PL at a high concentration.

Having a relatively low viscosity, CMP does not increase the rheological parameters of the PL gels. At low concentrations, CMP increases the T_gel_ and decreases the hardness, the complex viscosity and the viscoelastic moduli of the gels. Between 0.4% and 1% CMP, T_gel_ drops significantly while the gel hardness increases, but with a further increase in the CMP concentration, the gels were softened. The addition of CMP has two opposite effects on the thermally-induced gelation of PL: it increases the concentration of PL in the polyelectrolyte-free domains, decreasing the T_sol–gel_ and CMT and it perturbs the aggregation of PL micelles into an organized structure in the whole network, softening the gels. 

The DLS studies of concentrated PL solution proved to be able to evidence the formation of the micelles by a decrease in the decay time of the slow relaxation mode, and the aggregation of the micelles by the appearance of a power-low tail in the autocorrelation function. In the presence of CMP, the diffusion of the polyelectrolyte chains overlapped with the organization of the PL micelles, but the formation of hard gels can be evidenced by the appearance of a power-low in the intensity-time correlation function. 

The PL17/CMP gels showed a good recovery even after a strain of 1000%. The sample PL17/CMP_0.95_-0.4, which has the lowest rheological parameters due to the extended conformation of the polyelectrolyte in the PL network, achieved a higher degree of structure recovery after each strain cycle. 

In summary, the carboxymethylation of pullulan can improve its miscibility with PL, but high substitution degrees are required to obtain miscible polymeric mixtures. The addition of CMP (with DS = 1 or higher) modifies the strength of the PL thermogels depending on the polyelectrolyte concentration. However, the addition of 1% CMP to the PL 17% system led to the obtaining of thermogels with convenient parameters: T_gel_ around 26 °C and a good hardness.

## Figures and Tables

**Table 1:** Composition of some of the studied formulations.

Formulation Code	PL Solution Used for the Preparation of the Sample (%, wt:wt)	CMP_0.95_ Concentration (%, wt:wt)	Sample Composition		
			PL (%, wt:wt)	CMP_0.95_ (%, wt:wt)	Water (%, wt:wt)
PL17	17	0	17.0	0	83.0
PL17/CMP_0.95_-0.4		0.4	16.9	0.4	82.7
PL17/CMP_0.95_-1		1	16.8	1	82.2
PL17/CMP_0.95_-3		3	16.5	3	80.5

**Table 2:** Thermogelation parameters for PL17 and PL17/CMP_0.95_ mixtures determined from different measurements.

Formulation	Tube Inversion Method	Textural Analysis	Rheology						DLS	
	T_gel_ (°C)	Hardness ^(a)^ (N)	T_o_ (°C)	T_sol–gel_ (°C)	T_gel_ (°C)	G′ ^(b)^ (kPa)	G″ ^(b)^ (kPa)	η* ^(b)^ (kPa∙s)	CMT (°C)	T_gel_ (°C)
PL17	26.4 ±0.4	0.23 ± 0.014	22	22	26.9	11.2	3.1	11.6	16	21
PL17/CMP_0.95_-0.4	29.3 ± 1	0.12 ± 0.004	21.2	21.9	29.3	4.3	1.7	4.6	16	29
PL17/CMP_0.95_-1	25.2 ± 0.3	0.30 ± 0.018	20.8	21.4	25.8	7.6	2.7	8.1	15	26
PL17/CMP_0.95_-3	23.8 ± 0.4	0.16 ± 0.005	16.3	18.6	22.5	4.7	1.9	5.07	14	24

^(a)^ measured at 37 °C, ^(b)^ measured in gel state at 37 °C, ω = 1 rad/s, γ = 1%.

## Data Availability

Data are available on request.

## Supplementary Materials

The following supporting information can be downloaded at: https://www.mdpi.com/article/10.3390/polym15081909/s1, Figure S1: FT-IR spectra of pullulan, CMP_0.42_, CMP_0.95_ and CMP_1.6_; Figure S2: FT-IR spectra of CMP_0.95_, PL17 and PL17/CMP_0.95_ gels. References [20,28,84,85,86,87,88] are from the supplementary materials.
