# Supplementary material for: Poloxamer/Carboxymethyl Pullulan Aqueous Systems—Miscibility and Thermogelation Studies Using Viscometry, Rheology and Dynamic Light Scattering
Source: Polymers (Basel). 2023 Apr 16;15(8):1909. doi: 10.3390/polym15081909 (PMC10143542; doi:10.3390/polym15081909)
Supplement: Supplementary file 1 [file polymers-15-01909-s001.zip › polymers-2271232-supplementary.pdf]

Supporting Information

Poloxamer/carboxymethyl Pullulan Aqueous Systems-Miscibility and Thermogelation Studies using Viscometry, Rheology and Dynamic Light Scattering

Irina Popescu *, Marieta Constantin *, Maria Bercea, Bogdan-Paul Coșman, Dana Mihaela Suflet and Gheorghe Fundueanu

I. Structural characterization of Carboxymethyl pullulan

The degree of substitution (*DS*) of CMP_x, which corresponds to the number of carboxymethyl groups per anhydroglucose unit, was determined by conductometric titration according to Constantin et al. [20]. For this, 0.1 g of CMPx was dissolved in 25 mL of distilled water and 5 mL of NaOH 0.1025 N were added. This solution was titrated with hydrochloric acid 0.1025 N using a conductometer CMD 210 (Radiometer, Copenhagen, Denmark) equipped with a CDC 865 cell. From the titration data, the milliequivalents of -COONa groups per gram of CMPx were calculated using Eq. (1)

$$C(meq./g) = \frac{V_{HCl} \times c}{m} \quad (1)$$

where V_{HCl} represents the volume of HCl aqueous solution determined from the conductometric titration curve, c is the concentration of HCl solution (mol/L), and m is the mass of CMPx (g) used in titration.

DS value of CMPx was determined with Eq. (2):

$$DS = \frac{162 \times C}{162 + 80 \times C} \quad (2)$$

where C represents the content of carboxymethyl groups in equivalents/gram of polymer, *80* is the molecular mass of the carboxymethyl radical, and *162* represents the molecular mass of anhydroglycosidic unit.

FT-IR spectra were obtained in KBr, using a FT-IR Vertex 70 spectrometer (Bruker, Austria). The spectra of native pullulan and CMPx derivatives are presented in **Figure S1**. The characteristic absorption bands of pullulan are present in the FT-IR spectrum of CMPx. In addition, the appearance of new bands specific to newly introduced carboxymethyl groups, confirms the successful realization of the carboxymethylation reaction. The absorption bands observed at 1610 cm^{-1} and 1422 cm^{-1} highlight the antisymmetric and symmetric stretching vibrations of the C=O bond from the $–COO^-$ groups [84,85]. The shoulder at 1720 cm^{-1}, corresponding to the C=O bonds from COOH groups, is visible in the $CMP_{0.42}$ spectrum and hidden under the large band at 1610 cm^{-1} for the others two CMP derivatives spectra, showing that the carboxylic groups are in a large extend in the form of sodium salt. The other bands were asigned as follows: 3428 cm^{-1} (O-H stretching vibration), 2925 cm^{-1} (C-H stretching vibration of methylene groups), 1326 cm^{-1} (C-O stretching), 1157 cm^{-1} and 1020 cm^{-1} (C-O-C vibration and C-O stretching bands, respectively) [86], 852 cm^{-1} corresponding to α-configuration of α-D-glucopyranoside units [87] and those at 757 and 930 cm^{-1} indicated the α(1-4) and α(1-6) linkages, respectively [28]. The peaks at around 850 and 756 cm^{-1} show the pullulan's $^4C_1$ chair conformation. The reduced intensity and the shift towards to lower wavenumber of the absorption band at 3428 cm^{-1} due to the OH stretching with the increase of DS, indicates that some of OH groups were carboxymethylated. Also, with the increase in the –COONa groups number, the bands corresponding to the stretching vibration C=O of carboxylic groups increase in the intensity.

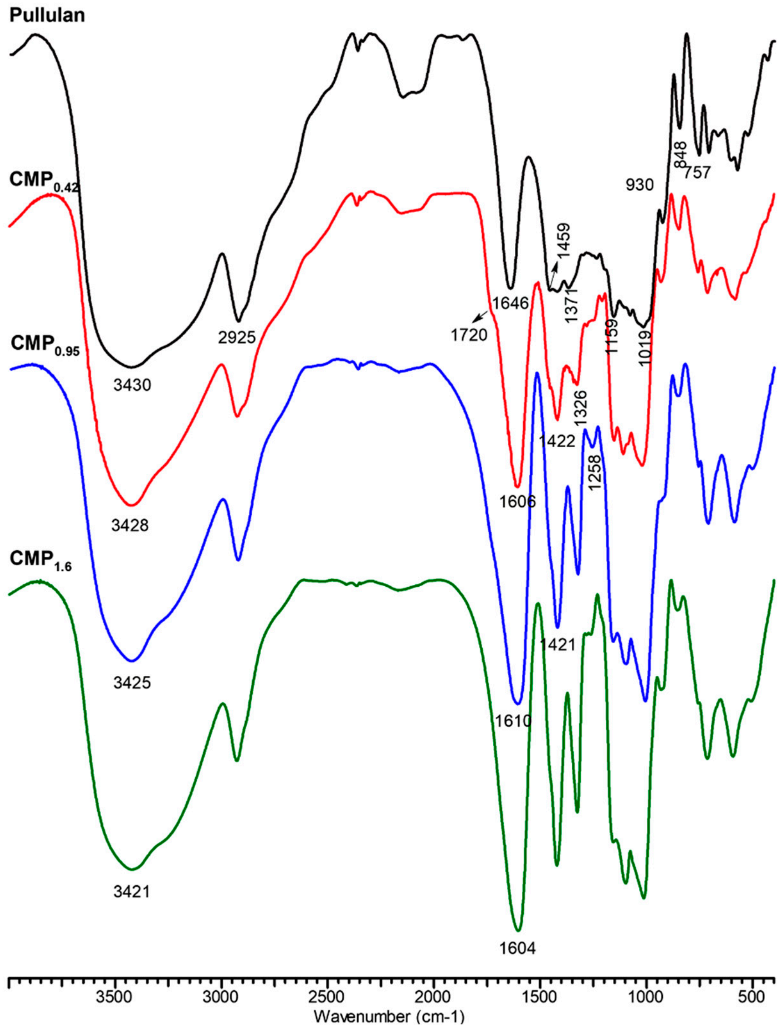

Figure S1. FT-IR spectra of pullulan, $CMP_{0.42}$, $CMP_{0.95}$ and $CMP_{1.6}$.

II. FTIR spectra of PL/CMP gels

The gels obtained at 37 °C were frozen in liquid nitrogen and dried by liophilization. The samples were mixed with KBr and pelletized for FT-IR spectroscopy. **Figure S2** presents the spectra of PL17, PL17/$CMP_{0.95}$-3 gels together with the FT-IR spectra of the $CMP_{0.95}$ derivative. The characteristic peaks for poloxamer, 2888 cm^{-1} (C-H stretching), 1343 cm^{-1} (O-H bending), 1110 cm^{-1} (C-O stretching), 1061 cm^{-1} and 1468 cm^{-1} (CH_2 scissor and CH_3 deformation vibration) [88] were present in the spectra of PL17 and PL17/$CMP_{0.95}$-3 samples. The presence of the band from 1610 cm^{-1} characteristic to carboxylate groups of CMP in the spectrum of PL17/$CMP_{0.95}$-3 sample is an indication of the absence of chemical interaction between poloxamer and CMP. The large band from 3425 cm^{-1} from the spectrum of $CMP_{0.95}$ derivative corresponding to the O-H stretching vibration was displaced at 3430 cm^{-1} in the mixture, suggesting the formation of hydrogen bonds, most probably between the –COOH groups of CMP and the oxygens from the poloxamer chains.

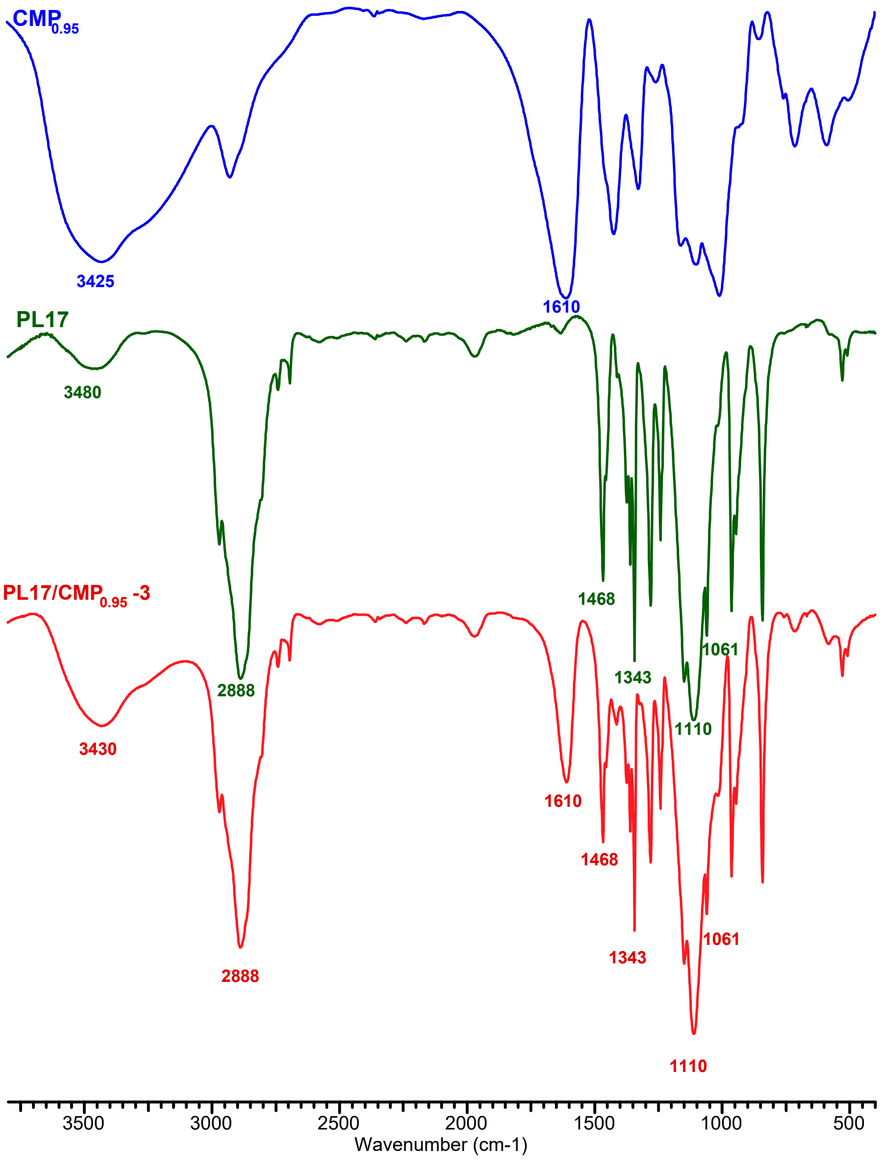

Figure S2. FT-IR spectra of $CMP_{0.95}$, PL17 and PL17/$CMP_{0.95}$ gels.

References:

20. Constantin, M.; Cosman, B.; Bercea, M.; Ailisei, G.L.; Fundueanu, G. Thermosensitive poloxamer-graft-carboxymethyl pullulan: a potential injectable hydrogel for drug delivery. *Polymers* **2021**, *13*, 3025. Doi: 10.3390/polym13183025

28. Singh, R.S.; Kaur, N.; Singh, D.; Kennedy, J.F. Investigating aqueous phase separation of pullulan from Aureobasidium pullulans and its characterization. *Carbohydr. Polym*. **2019**, *223*, 115103. https://doi.org/10.1016/j.carbpol.2019.115103

84. Ellerbrock, R.H.; Gerke, H.H. FTIR spectral band shifts explained by OM-cation interactions. *J. Plant Nutr. Soil Sci.* **2021**, *184*, 388-397. Doi: 10.1002/jpln.202100056

85. Navarrete, J.T.L.; Hernandez, V.; Ramirez, F.J. IR and Raman Spectra of L-aspartic acid and isotopic derivatives. *Biopolymers* **1994**, *34*, 1065-1077. Doi: 10.1002/bip.360340810

86. Shingel, K.I. Determination of structural peculiarities of dextran, pullulan and gamma-irradiated pullulan by Fourier-transform IR spectroscopy. *Carbohydr. Res*. **2002**, *337*, 1445-1451. Doi: 10.1016/s0008-6215(02)00209-4

87. Thirumavalavan, K.; Manikkadan, T.R.; Dhanasekar, R. Pullulan production from coconut by-products by Aureobasidium pullulans. *Afr. J. Biotechnol*. **2009**, *8*, 254-258.

88. Karolewicz, B.; Gajda, M.; Gorniak, A.; Owczarek, A.; Mucha, I. Pluronic F127 as a suitable carrier for preparing the imatinib base solid dispersions and its potential in development of a modified release dosage forms. *J. Therm. Anal. Calorim*. **2017**, *10*, 383–390. Doi: 10.1007/s10973-017-6139-1
